# Book Reviews

**Published:** 1814-05

**Authors:** 


### CRITICAL ANALYSIS

### *OF RECENT PUBLICATIONS*

IN THE

### DIFFERENT BRANCHES OF PHYSIC, SURGERY, AND MEDICAL PHILOSOPHY.

*Commentaries on the Treatment of the Venereal Disease, particularly in its exasperated State; including a second Edition of a former Publication on that Subject, considerably augmented and improved. On the Use of Mercury, so as to ensure its successful Effect. With an Appendix, on Strictures of the Urethra, and on Morbid Retention of Urine.* By EDWARD GEOGHEGAN, Member of the Royal College of Surgeons in Ireland, Honorary Member of the Royal Medical Society, Edinburgh, and of the Physico-Chirurgical Society, Dublin. 8vo. pp. 219. Cumming and Co. Dublin, 1814.

THIS appears to be the production of an accurate and experienced observer, and will be found to contain much information of importance to the practitioner. To the surgeons in the army and navy, and especially to the junior part of them, this work will prove a great acquisition. No mistake is more common than the reputed facility of curing venereal disease. The most ignorant pretender to the science of medicine, if equipped with a sufficient quantity of mercurial ointment, never dreams of the possibility of failure in the cure of one of the greatest ills that mortals can endure. The fact in reality is far different. Mercury is only a specific when properly applied, and becomes a most destructive poison when administered without regard to the constitution of the patient, or the particular state of the symptoms. To Mr. G. the profession will feel indebted for having defined, in a more satisfactory manner than has hitherto been done, the limits of the *mercurial treatment;* and for having pointed out the distinction between the essential symptoms of the disease, and those that are merely accessory to it, and dependant on the contingencies of the moment.

The preface announces it to be a second and corrected edition of a work which appeared in 1802, with additional observations on the treatment of the venereal disease, especially on the means of preventing the destruction of the uvula and palate when ulcerated, and on the use of mercury so as to ensure its successful effect. Mr. G. after stating the objects of the work, adverts to the particular opinions of some writers on this subject.

"In the course of my observations (he remarks) I shall have occasion to animadvert on the opinions of the most respectable authorities, particularly the late John Hunter, whose splendid talents, and unceasing labours for the improvement of his profession, furnish a noble example, and entitle his memory to veneration: it is on practical points chiefly that I differ with him, and I hold it a duty to contest whatever appears to me to be erroneous in this respect. Some of the speculative opinions of Mr. Abernethy strike me as not satisfactory; however, his practical rules furnish valuable guides, and cannot be too strongly inculcated. Whilst I consider Mr. Hunter's Treatise as abounding with suggestions of great value to the experienced pratitioner, I by no means esteem it a safe guide to the inexperienced.

"The difficulties with which the treatment of the venereal disease is so often embarrassed, invite still farther discussion, and require the application of the best talents and information in the medical profession. It has always excited my surprise that the physician directs his attention so little to this disease, and that systems of medicine refer to surgical writers for a particular account of it: its treatment certainly includes much surgery, and he who is the best medico-chirurgeon, will be the most competent to it. It is deeply to be regretted that so much of the practice devolves on unqualified persons, as there is no affliction to which human infirmity is liable, the effectual cure of which is so very important."

The author proposes to consider some symptoms attendant on the venereal disease, the nature and treatment of which, he thinks, are not well understood; and he is the more desirous of entering into the investigation, finding that the most respectable modern authorities are not only undecided in their opinions, but inculcate a practice which appeared to him highly injudicious, and from which he has witnessed the most destructive consequences. He observes,

"Although every form the venereal disease exhibits, furnishes ample matter for observation, I shall confine myself to some aggravated symptoms, in the treatment of which I have had considerable experience, and which have given rise to these reflections. I particularly allude to phymosis in the inflammatory stage, and to phagedenic chancre; and I know of no symptom the event of which is more interesting, as they frequently terminate in the destruction of a part or of the entire penis. For some time back my attention has been directed to this point, but the number of cases which occurred during the summer, autumn, and winter of 1799, particularly excited my astonishment; and on communicating with other practitioners, I found that they met with similar instances in a far greater number during the same time, than at any former period: as to the nature and treatment of the complaint, the opinions generally entertained were different from those I had formed.

"It was remarked by the public, that the venereal disease then raging appeared to be singularly malignant; and I have heard even practitioners say, that they thought there was an unusual degree of virulence in the infection. The appearance which gave rise to these remarks, was violent tumefaction of the penis, often terminating in mortification, particularly when injudiciously treated: other symptoms were also observable, as singular for intensity of degree."

Again,

"When the ordinary symptoms of an infectious disease appear to be exasperated in an unusual degree, the question arises, to what are we to attribute this increased degree: whether to increased acrimony of the poison, or to any adventitious or physical causes, insensibly operating? This is the pivot upon which the point of practice must turn. If to the former, mercury is the remedy; but if to the latter, many and various circumstances are to be taken into consideration, which are too frequently overlooked. There is nothing more common than to attribute those venereal appearances which resist the effects of mercury, or are increased while it is administered, to an original morbid condition of the habit; and the plan of treatment is the administration of bark, opium, wine, to which mercury is conjoined by some. Decoctions of the woods, and sea bathing, are also very much used; and these means are recommended by authors, and very generally pursued in a kind of routine, as if they had a specific operation in all diseases which had a venereal origin."

He adverts to the opinion and practice of Mr. John Hunter respecting phymosis,—" 'that when this tumefaction takes place, in consequence of a chancre, he suspects there is an irritable disposition in the habit, for it is plain there is more than the specific action, the inflammation extending beyond the specific distance.' In his direction for the constitutional treatment, he seems a good deal perplexed: his words are, 'In those cases where violent inflammation has attacked the seat of a chancre, producing phymosis, as before described, and often so as to threaten mortification, a question arises,—is mercury to be given freely, to get rid of the first cause? Nothing but experience can determine this.'" Mr. Hunter then proceeds to recommend mercury given *sparingly*, bark, opium, but in so equivocal and inconsistent ent a manner as to leave us without any fixed principle as to the nature of the complaint, or the mode of treating it. He also advises, in the local treatment, to inject mercurials, even corrosive sublimate, in the proportion of one grain to an ounce of water, and other mercurials, inside the prepuce, to remain in contact with the parts; but concludes by saying that he has his *doubts* as to the propriety of using any irritating applications in such cases.

After examining the question whether increased acrimony of the poison has any share in producing these aggravated symptoms of the complaint, he concludes with the inference "that mild or violent symptoms, whether accompanied by inflammation or ulceration, or in whatever form they appear, are not characteristic of the degree of acrimony in the infectious matter. Hence we have no reason for attributing the aggravated state to the infectious matter alone: we are led then to look for an explanation of the phenomenon from some other cause. It is a common phrase, when things run untowardly, to say, this is owing to *peculiarity of constitution;* but in what this peculiarity consists we are uninformed, and, of course, are without any guide as to the treatment."

In considering the human constitution, he observes, the great variety of circumstances which influence it every moment should be taken into account, the state of the air, place of residence, intemperance, effects of the passions, &c.

"Many alterations may take place in the constitution, during the treatment of the venereal disease, from some of the causes enumerated, in which state mercury would be contra-indicated. When the penis becomes the seat of disease, its sensibility is preternaturally increased. Should any additional cause of disease operate locally, or generally, at the same time, it is obvious that the diseased part will feel its effects in a greater degree than any other, and inflammation be produced, which may take place whether chancre exists or not, as in gonorrhea; and chancres may spread and exhibit the most malignant features, independently of the virus. In farther illustration, I will suppose a case of chancre attended with slight symptoms, and that by accident the part is suddenly struck, and violent symptoms ensued: are we not to judge of the latter, *quoad injuriam,* and would it not be error in the extreme to treat this case as venereal during the recent symptoms? And is it not manifest that the same effects may be produced through the medium of the constitution; do we not every day see the most violent diseases come on suddenly from an accession of cold, and affecting particularly such parts as were previously in a morbid state?"

In page 34, a paper of M. Brugerius, formerly surgeon in chief to the army of Italy, is quoted.

"'The military hospital of Toulon, the situation of which is low and close, proved formerly little better than a tomb for most of the

 patients patients admitted, and especially such as laboured under syphilis, the greater number of inflammatory venereal symptoms terminating in gangrene. Phymosis and paraphymosis were often followed by a total loss of the penis, the mortification sometimes spreading into the neighbouring parts. Gangrene also frequently seized on ulcerated buboes, spreading along the thigh, or the abdomen, and sometimes destroying all the external parts of generation. Struck with the alarming appearances which no art could remedy, M. Brugerius visited the military hospitals of the North, for the purpose of observing whether the venereal disorders in them were subject to similar accidents. The contrary was found to be the case, and M. B. therefore justly attributed their occurrence at Toulon to local causes. In fact, when the wards of the hospital were raised, and ventilation strictly attended to, these gangrenes ceased to make their appearance;' a striking proof of the great importance of pure air in the treatment of diseases."

At the time the symptoms referred to were witnessed in the highest possible degree, epidemics of the worst kind were extremely prevalent.

"It is admitted that inflammation of the penis, such as I describe, is erysipelatous, and we know that erysipelas is an usual attendant upon epidemic causes; and when it is considered that venereal patients are very numerous, and that they are very much exposed to the weather, surely when many diseases are produced, and all are aggravated by the state of the atmosphere, it is a fair induction that a number of persons labouring under the venereal disease will be affected by the prevailing epidemic, and that it will produce its effects as before explained, namely, by inflaming those parts which were previously in a morbid state. In this way I think that the exasperated symptoms so freqnent in the year 1799 may be accounted for. I have been informed by Mr. Henthorn, senior surgeon to the hospital called the Lock, that an extraordinary number of these cases presented themselves there at this period, but that they were of the putrid type, particularly among females; mortifications were very common, set in early, and often proved fatal."

"Local irritation also has a great share in producing those attacks: When we consider the high degree of sensibility of the penis, and this condition morbidly increased by the poison, and its being pendulous, and very liable to motion, it is obvious that it must be often irritated by striking against the breeches, &c. &c. and when the sores are small, little attention is paid to the means of obviating such injuries. Suppose one of the fingers having some small ulcers, although free from virulence, and that it was unprotected and pendulous, without bandage or dressing, coming in contact with foreign bodies, surely diffused inflammation might reasonably be expected. How much more susceptible is the penis of injury from a similar cause?"

A case is related of

"A young man who was using mercury for chancres, and when they were nearly healed, a dressing of the ung. eruginis was applied to to a small sore that proved obstinate: inflammation succeeded, the dose of mercury was increased, mortification took place. Two surgeons of great experience were employed: they advised calcined mercury to be given instead of the ointment; the mischief increased, all the neighbouring parts were destroyed, and it proved fatal. Correctness requires that I should mention he was ordered bark, opium, cicuta, &c."

"About the same time I met with several cases in which the sores were trivial, with every appearance of their being immediately healed, the habit fully under the influence of mercury, when unaccountably and suddenly the penis became greatly tumefied, and all *those* who persevered in the use of mercury, or took bark and wine, suffered a rapid destruction of parts. I remarked that almost all these patients were exposed to the weather, and some of them to great exercise. In one case it was produced by the application of a strong solutionof corrosive muriate of mercury, to remove warts. In another the inflammation had set in but thirty-six hours, and the penis was completely sphacelated when I first saw him. This patient was using mercurial frictions, and allowed to drink wine, and pursue what is called the invigorating plan, whilst in this state. He was of a robust habit, and in the twenty-first year of his age, and had considerable symptomatic fever. I directed that the mercury should be discontinued, and reversed the treatment, on which the general and local symptoms yielded; but it was too late to save the penis, a great portion of it having separated. In many instances delirium and considerable fever attended, &c."

In the treatment of phymosis, he proceeds on the principle that the inflammation exceeds that which the *venereal* virus usually produces,—that it is not *venereal*, as it is admitted that when an accessory disease takes place, it ought to be removed previously to attempting the cure of the original. Every principle of the healing art requires that this new disease should be first attended to.

"Surely were fever, pneumonia, catarrh, cynanche tonsillaris, to attack a patient known to be infected with the venereal disease, the mode of treatment in these diseases would be pursued, but no mercury, until after they had subsided."

The use of mercury is therefore very properly prohibited where active inflammation is present in parts of loose structure. In a case, related by Hunter, of its use in a sore throat mistaken for venereal, it produced mortification as soon as it affected the mouth.

"We every day see that in the mouth it produces violent inflammation, ulceration, and sometimes mortification. Granting that these consequences sometimes attend its use, it is reasonably to be dreaded that it will precipitate inflamed parts, of such structure as the penis, into mortification, and prove injurious in diseases of other parts, mistaken for venereal."

The

The treatment recommended if the symptoms run high, consists in bleeding, purging, and the antiphlogistic regimen. The use of bark and mercury, advised by Hunter, is objected to, we think upon strong grounds. Where phymosis occurs when the venereal sores are healing under the use of mercury, and where suspicion exists of the symptoms having arisen from the noxious effect of the mineral, bleeding is seldom necessary. A discontinuance of the mercury, with good air, is all that in general is required; in cases of debility, bark may be used.

Speaking of the local treatment, he condemns the use of leeches, which he thinks infinitely less useful than general bleeding, fomentations of liq. plumbi dilut. or an ointment of acetate of lead.

"Some surgeons recommend that the prepuce be slit up, as remedial of violent phymosis; in deciding on this practice, I would make this distinction: if the subject was very irritable, or unhealthy, I would disapprove of it; but if the contrary state prevailed, it might be done with advantage and without danger in some cases.

"In Chronic Phymosis, this operation is scarcely ever necessary; the closest contraction will yield almost always to bathing in warm milk twice or thrice a day, endeavouring each time to draw back the prepuce, and to force the glans through it, which acting as a wedge, by perseverance will gradually dilate the contracted prepuce.

"A case occurred some time ago, which will make me cautious as to operating on this affection." After the ineffectual use of lotions, &c. at the entreaty of the patient the operation was performed. During three or four days, nothing remarkable occurred; "he lived out of town, and walked in and out every day, a distance of one mile and a half; on the fourth or fifth day, he was exposed to a shower of rain, and ran some distance, so as to irritate the wound; inflammation and mortification took place within twenty-four hours; when I saw him, it had every appearance of extending, and was accompanied by fever, his eyes were highly tinged with yellow, and his tongue furred; I directed a scruple of jalap and three grains of calomel every day, so that four or five stools would be procured; the evacuations were highly bilious, he took no other medicine; a poultice of oatmeal and beer was applied, the integuments of the penis and scrotum sloughed off; and I observed, that as his bilious complaint grew better, a favourable change took place in the local affection: by a perseverance in purgatives he recovered. Here was a case of mortified penis, arising solely from external irritation, aggravated by a deranged state of the hepatic system, and cured by remedying the general condition of the body; had I followed the advice given in books, bark and wine, opium, &c. would have been the medicines, and especially as he was debilitated from age, original habit, and disease; and had he been young, how many would have contended that the venereal virus must have had a share in such a diseased penis."

It is right to observe, the opposite practice, which we consider to be extremely injudicious, is taught by Hunter, Howard, and Swediaur; it is therefore proper to counteract the influence of their authority on the junior part of our profession. The author next details the mode of treatment when sloughing of the prepuce has taken place, for which we refer to the work itself.

"Phagedenic Chancre is said to be characterised by the successive formation of sloughs, so as to destroy the part on which it is situated; when it occurs in patients labouring under the venereal disease, there is reason to fear that it may be treated injudiciously, in consequence of the opinion advanced by some, that it is occasioned by unusual virulence of the infectious matter; this view of the complaint would naturally lead to the exhibition of the antidote to the infection, in increased quantity."

The principle of practice, in this case, like the preceding, is from the fact, that

"Phagedenic chancre is not a truly venereal ulcer, but a *supervening* disease, and that the habit liable to it is apt to suffer materially from the use of mercury; an high degree of morbid sensibility is manifest in every such subject, which condition is invariably increased by mercury.

"Some surgeons discontinue it for a time, and resume it on a change of appearances; in the opinion that it is dangerous long to withhold the venereal antidote."

To which practice he objects, for several reasons detailed.

"When ulcers on the penis exhibit this character, the source will always be found in a morbid condition of the habit, or in local irritation, often in both conjointly; at the commencement, before mercury has been used, evacuations are indicated, and a strict avoidance of irritation of every kind; at this period, bark and the tonic plan are contra-indicated, yet they are very much recommended, on the appearance of a slough, and if a favourable change did not soon take place, opium, cicuta, nay, even mercury, are given. When it is considered that a change of the wind, mental excitement, intemperance, or any derangement, are sufficient to occasion a sloughy state, we should hesitate before we give astringents, or narcotics; the latter are particularly exceptionable in the early stage of the complaint, and should not be persevered in, at any period, or in any case, if a favourable change did not soon succeed their exhibition, because, being possessed of deleterious properties, they must prove prejudicial, if unattended with salutary effects; cases are recorded, in which cicuta was pushed to great extent, with the worst consequences, yet the mischief was attributed to the disease, instead of the intended remedy."

"Burrowing sores partake of the nature of phagedenic. Mr. Abernethy says, decidedly, that these are not venereal, in which opinion I agree with him, but to a limited extent; namely, that they are are not perpetuated by the venereal poison, and that they are aggravated by mercury, and may be produced by it. That sores which burrow, situated in the neighbourhood of the genitals, occur sometimes, without a venereal origin, I am satisfied.

"Having decided that the restoration of the health is the chief indication, the means of effecting this is particularly to be attended to. When phagedena, or a burrowing sore, is an early symptom, it is almost invariably accompanied by quick pulse, dry skin, furred tongue, and great pain; hence the necessity of purging until the tongue becomes clean, and the anodyne and sudorific medicines, as directed in treating of phymosis; if the distress is excessive, bleeding in proportion to the strength, will be advisable at this early period; and in the more advanced stage, warm baths, and the soothing plan. In obstinate cases, a dry and warm atmosphere, particularly in the country, will be of material service; the only medicine I can recommend from experience, is sarsaparilla given alone; when it is combined with guaiacum, and the other ingredients that form the decoctum lignorum, the habit is too much excited, an effect that is unfriendly to an irritable condition. I also object to mercury in any form accompanying sarsaparilla, on the same principle, and also for the reasons advanced before."

"As to the local remedies, my experience does not warrant me in saying much; the fermenting poultice of so large a size as to embrace the entire penis, is a good application in many cases; however, it is sometimes found too irritating, and its weight is distressing; under these circumstances, an ointment composed of one part of the ung. elemi and four of the ung. ceræ, may be substituted with advantage; it is a good mode of applying it, to melt it in a spoon, and pour it into the sores; this is adapted to the sloughy state. In obstinate sores that do not slough, I use the ung. hydrarg. nitrat. mixed with six parts of the ung. ceræ; applications that are more stimulating, are apt to prove mischievous in all sores of this part; these ointments should be blended together, with the assistance of heat."

"I believe it is the usual course of practice, to commence the administration of mercury, after the exasperated symptoms had subsided, so that time should not be lost in subduing the virus; to which my experience obliges me refusing assent, having met several cases, in which the mischief was renewed with increased violence, in consequence of this practice; a case has been communicated to me lately, of a person labouring under a foul ulcer of the penis, in the treatment of which the surgeon discontinued the use of mercury, until the sore became the size of a pea, it was then thought advisable to give mercury; on its administration, the features of the sore immediately altered, and it spread so as to destroy a great part of the penis. It has been a rule with me, during the last twelve years, never to resume the use of mercury in those cases, until after the sore was cicatrized; and in subjects with whom mercury disagreed very much, I have withheld it, and waited for constitutional symptoms, and in some instances the disease never returned, which I attributed to the removal of the virus *in limine*, by the sloughing."

There

There is no symptom of the venereal disease so embarrassing as a rapidly increasing ulceration of the fauces, with sloughing of the soft parts, and destruction of the palate and bones of the nose. We have now, in our own practice, three cases of this affection produced by the action of mercury administered for disease supposed to be venereal. In all these more or less of the bones of the jaw and palate have been removed in large portions. It becomes then of great importance to be able to distinguish that which is essential to the disease from that which is the effect of the remedy.

"A young gentleman of the medical profession laboured under an ulcerated bubo, which spread considerably under the use of mercurial frictions. They were discontinued, and the sore amended gradually. After a few months it healed, and he removed to the country, where the throat became ulcerated, accompanied by febrile symptoms. The means of relief pursued having proved ineffectual, I was brought to see him, and found him greatly emaciated, pulse small and quick, and skin parched; the uvula was so relaxed as to impede deglutition, its edges ulcerated; no benefit was derived from gargles. The seat of the mischief seemed to me to be out of the reach of applications in the usual way, and I considered the case venereal. I ordered a lotion composed of two grains of muriate of mercury dissolved in seven ounces of water, and an ounce of mel. rosæ, to be snuffed up the nostrils, until it reached the pharynx; then to be ejected through the mouth; and half a grain of calomel, and a grain of antimonial powder, to be taken night and morning. This practice soon proved successful, and he took no other medicine for six or seven weeks; the quantity of calomel was increased, after the constitution became habituated to it. On discontinuing the pills, he commenced the use of sarsaparilla, which he continued about a month; and has remained free from disease ever since, about seven years."

(To be continued.)

---

*Expériences sur le Principe de la Vie, notamment sur celui des mouvemens du Cœur, et sur le Siége de ce Principe, suivies du Rapport fait à la Première Classe de l'Institut sur celles relatives aux mouvemens du Cœur.* Par M. Le Gallois, Doctor en Medicine de la Faculté de Paris, &c. &c. pp. 368. 8vo. Paris, 1812.

The author begins an introductory section, by stating that sensation and motion have generally been considered as the characteristic properties of a living animal body. He then proceeds to give an historical sketch of the opinions which have been entertained respecting the part of an animal which is necessary to the existence of these faculties, or, in other words, the seat of the vital principle. At first view, these properties might be supposed inherent in every part of the living body, since each appears to possess them to a greater or less extent; but experience having taught us that the division of a nerve deprives the parts to which its inferior extremity is distributed, of sensation and motion, we must conclude, that sensation does not reside in the part which feels, nor motion originate in that which moves. Hence, by tracing the nerves to their origin, the brain naturally came to be looked upon as the focus of the nervous power, and the seat of the vital principle, and many facts combined to favour this idea. Any considerable injury to the brain produces instant death; and when the spinal marrow or a nerve is divided, the parts whose nerves communicate with the brain continue to move and feel, whilst those below are paralysed.

Other inquirers, having observed that some parts of the brain might be injured or destroyed with impunity, entered upon an ineffectual search after the part of the brain immediately necessary to life,—the sensorium commune, or seat of the soul. But the opinion that even the whole brain was the exclusive seat of the vital principle, was not easily reconcilable with some well-ascertained facts. Why do cold-blooded animals continue to live for months after the removal of their heads; or why should this term vary according to the manner in which the brain has been extracted? It is not sufficient to say that the laws of the animal economy are different in cold and warm blooded animals, for similar facts have been observed with respect to this latter class.

The author here refers to many respectable authorities, among whom are Kaaw, Boerhaave, Lamitrie, Cuvier, and Haller, for instances of warm-blooded animals continuing to move after decapitation; and adduces the fact of acephali arriving at a full growth, and even living for some days after birth, as an additional proof of the inaccuracy of this opinion.

Haller's theory of irritability did not satisfactorily account for these phenomena. His experiments went to prove that the condition necessary to motion exists in the muscular fibre itself; that in the voluntary muscles, the nervous power is the stimulus to contraction, but in the other moving parts, particularly the vital organs, the stimulus is of an entirely different nature. He conceived that the internal vital functions might be carried on independently of the nervous power, so long as they were able to dispense with those functions which are under the influence of the will, and of course of the nerves, particularly respiration. Now as the fœtus does not breathe, and as cold-blooded animals are able to survive a long privation of air, and both preserve their irritability during a considerable period, it followed that the nervous influence was not in them necessary to life.

Mons.

Mons. le Gallois points out a few decisive objections to this doctrine. Voluntary motion takes place in acephali and decapitated frogs; and Fontana's experiment of preserving life by artificial inflation of the lungs, shows that the same thing may occur in warm-blooded animals. These motions cannot be referred to irritability, which term is used to imply the contractile faculty which a muscle possesses, and not the stimulus which disposes it to contract. If we remove the leg of a frog, and irritate a nerve or a muscle; in the first case all the muscles to which that nerve is distributed, in the second case the muscle itself, will be thrown into a state of contraction. These phenomena are always observable a certain time after death. But the decapitated frog is a living animal, and exhibits very different phenomena. If scratched with a pin, he feels, and expresses his sensation by moving all the voluntary muscles.

Having premised these observations, Mons. le Gallois enters upon his first memoir. A case of difficult labour which occurred to him, first attracted his attention to the period during which an infant may live without breathing after all communication with the mother has ceased. A series of experiments upon this subject led him to the conclusion, that animals support the privation of air for a time which bears an inverse proportion to their age. A rabbit, immediately after birth, will retain sensation and motion for fifteen minutes after it has been entirely deprived of air; whilst another, a month old, will not retain them longer than two minutes under the same circumstances. The author observed that decapitated animals remained alive during the same length of time as animals of the same age and species did, when totally deprived of air. There was, however, one obvious difference between the two cases: the strangled animal made violent efforts to draw in air; the decapitated animal made none. In the former case, these efforts, together with gaspings (baillemens), were the last signs of life, and remained after the extinction of sensation and voluntary motion; in the latter case, the same gaspings occurred, but the muscles of respiration were perfectly paralysed from the first instant. He divided the spinal marrow between the occiput and first vertebra, and the effects were precisely similar to those which succeed decapitation. Hence he concluded, that the decapitated animal dies from strangulation, and that to preserve life, it was but necessary to keep up respiration by the artificial inflation of the lungs; and this conjecture was verified by the result of the experiment. M. le Gallois has, however, been anticipated in this discovery by Fontana and Chirac, the latter of whom in particular de-

 scribes scribes the same means of preserving the life of an animal after decapitation.*

Having thus ascertained that the brain is not necessary to sensation and motion, the author, after decapitating a rabbit, whilst keeping up artificial respiration, destroyed the spinal marrow by passing an iron stilet along the vertebral canal. Every sign of life instantly disappeared, and irritability alone remained, as is always the case during a certain time after death. In another rabbit, he destroyed the spinal marrow, without removing the head: the result was exactly similar to that of the preceding experiment, except that the gaspings (baillemens) were observed in the head. He divided different animals transversely into two portions, and found the signs of life to remain in both parts, during a period which varied inversely with the age of the animal. On destroying the spinal marrow in either of these portions, it immediately lost every appearance of life, whilst the other continued to move. If the spinal marrow was partially destroyed, the parts which received their nerves from the destroyed portion lost the signs of life immediately. To prove that the signs of life in these cases do not depend upon any of the thoracic or abdominal viscera, but entirely upon the spinal marrow, M. le Gallois removed all the viscera from these cavities, and then separated the head: the animal continued to live, but the moment that the spinal marrow was destroyed, death ensued.

The conclusion to be drawn from these experiments is, that life may exist independently of the brain, and of the viscera of the thorax and abdomen, though these are certainly necessary to its continuance. The author proceeds to inquire in what this necessity consists. The motions of respiration depend upon the brain, through the phrenic nerves, and those which supply the other respiratory muscles arise from the spinal marrow; the motions subservient to respiration cease when the connexion between the origin of these nerves and the brain is divided; whilst other parts deriving their nerves from the spinal marrow continue to move. The author thinks that the cause of this singular fact is in some manner connected with the course of the spinal accessory nerve. To determine in what part of the brain the influence which governs the motions of respiration is situated, he opened the cranium of a rabbit, and removed the brain by successive layers. He thus removed the whole of the cerebrum

* The writer of this article is not in possession of correct information relative to Fontana's experiments, and the lateness of the month at which this is sent to press prevents the necessary investigation. The account, however, of Chirac's will be found in the *Journal des Sçavans*, and in the *Collection Academique*, from which it is transcribed.

cerebrum and cerebellum, and a part of the medulla oblongata. Respiration went on till that portion of the medulla oblongata where the eighth pair of nerves arise was cut away; it then ceased. By performing the operation in such a manner as to preserve this part, he was enabled to preserve animals alive after decapitation, without the aid of artificial respiration; but from the hæmorrhage, and the deficiency of circulation in the extremity, together with the diseased state of the part arising from its exposure, warm-blooded animals never survive longer than half an hour. These obstacles were not met with in cold-blooded animals to the same extent, whilst the length of time which they are able to exist without food, rendered them particularly well adapted for this experiment. They were found to survive decapitation performed in this manner during several months. Some lizards, in which this part of the medulla oblongata was removed, lived longer than when totally deprived of air, which is attributed by the author to a process analogous to respiration being carried on through the skin. The brain is also necessary to the permanence of the life of the trunk, by the influence which it exerts through the par vagum; but this inquiry forms the subject of the second memoir. The abdominal viscera, the lungs, and the heart, are necessary to the formation and circulation of arterial blood. Life, the author says, does not depend on circulation, for sensation and motion remain after the heart is removed. But this experiment can hardly be considered as conclusive, as Bichat* informs us that he has seen the circulation carried on in the capillary vessels of frogs, by what he calls their tenacity, after the heart was removed. M. le Gallois supposes that the impression communicated by the arterial blood preserves the nervous organs in a state of energy during a variable period, after which the impression requires to be repeated. He found, after tying the inferior aorta in a rabbit, that motion and sensation ceased in its hinder parts.

The subject of the second memoir is the investigation of the seat of the principle which governs the motions of the heart. M. le Gallois concluded, from the experiments already detailed, that two conditions were necessary to preserve life in any part of an animal body: 1st, the integrity of a corresponding portion of the spinal marrow, and its nervous communications; 2ndly, the circulation of arterial blood through that portion. He destroyed the lumbar portion of the spinal marrow in a rabbit two days old: the posterior extremity lost every mark of life, whilst the anterior part remained alive; but after the lapse of three minutes and a half, every sign of life became extinct. He repeated the

* Recherches Phys. sur la vie et la mort, p. 150.

experiment experiment several times, and practised pulmonary inflation in some instances, but the same result constantly recurred. The destruction of the dorsal or cervical portions was followed by death after a still shorter period. Younger animals were able to lose a larger portion of the marrow without death ensuing, but the destruction of a considerable portion of it at once, proved fatal in every instance.

These facts pointed out two distinct sorts of influence which a part of the spinal marrow exerts over the living body: the first, that immediate influence which it possesses over the parts to which the nerves which arise from it are distributed, which immediately die when it is destroyed; the second is that by the destruction of which universal death is occasioned a few minutes afterwards. If the author's conclusions from his former experiments were correct, the destruction of one portion of the spinal marrow could only destroy the life of parts which received their nerves from other portions, either by injuring the integrity of the other portions, or by causing a cessation of the circulation of arterial blood through them. To bring this point to the test, he divided the spinal marrow transversely, in a rabbit of twenty days old, between the lumbar and dorsal vertebræ: sensation and motion continued in both extremities, but on irritating one, no motion was produced in the other. The anterior part of the animal did not appear to feel, when the posterior part was irritated. Strong convulsions were produced in the hind parts when the lumbar portion was destroyed, whilst the anterior parts did not appear at all affected by it. Nevertheless, general death took place three minutes afterwards. The cause was then to be sought for in some derangement of the circulation. Haller's opinions respecting the motions of the heart have been generally adopted. He supposed that the heart, like other muscles, possessed irritability; and that the stimulus which excited its contractions was the blood, independent of nervous influence. When this exciting cause was present in the ventricles, they contracted and expelled it; then relaxing from the absence of the stimulus, a fresh supply was poured in from the auricles. In support of this doctrine, it was argued, that the motions of the heart are independent of the brain, which was supposed to be the sole focus of the nervous power. It was also alledged that if the heart be removed from the body and placed upon a table, its contractions continue. This is true, but it remained to be ascertained whether these contractions possessed sufficient force to keep up the circulation. The signs of circulation are, principally, scarlet hæmorrhage on the amputation of a limb, and the red colour and fulness of the carotid arteries; but these are frequently equivocal. The

2 gaspings gaspings which take place after the removal of the head have a determined period in animals of the same age, and this period coincides with that which they observe after circulation has ceased in the head. By these means the author could ascertain very exactly the moment at which circulation stopped. He next gives the minute details of a part of the numerous experiments which he made, by destroying different parts of the spinal marrow in rabbits of different ages, and noticing the effects upon the circulation. The destruction of the cervical portion proved more generally and instantaneously fatal than either that of the dorsal or lumbar portions. After the age of twenty days, the destruction of any of the three portions proved fatal by stopping the heart's action in about three minutes. In some of the cases, artificial respiration produced an appearance of scarlet blood in the carotid artery, after every other sign of circulation had disappeared. This occurs in those cases in which the destruction of the spinal marrow is commenced at the upper extremity, when strangulation having taken place before the cessation of the heart's action, the pulmonary veins and left side of the heart contain dark blood. If pulmonary inflation be practised under these circumstances, it will change the colour of the blood as far as the carotid arteries, after circulation has entirely ceased. To prove this, M. le Gallois took two rabbits, one of which he killed by forcing a stilet through the brain and the whole spinal cavity, the other by strangulation. Forty-five minutes afterwards he opened their chests, and having ascertained that the blood in the pulmonary veins and left auricle was black, and that the heart exhibited no signs of irritability when scratched with a scalpel, he practised pulmonary inflation. The blood became of a bright scarlet colour in the pulmonary veins and left auricle, but it does not appear that any of this blood reached the carotid arteries, though it probably would have done so, had the motions of irritability continued.

Having thus ascertained that the effect of the destruction of one portion of the spinal marrow upon other portions is by cutting off their supply of arterial blood, the author proceeds to state some other facts respecting the destruction of the marrow. He found that when he confined, by means of ligatures, the sphere of the circulation within narrow limits, the animal was able to bear the loss of a much larger part of the spinal marrow, without the action of the heart becoming too weak to carry on the circulation. If the spinal marrow was destroyed gradually by small portions, the same result was obtained, which the author attributes to the death of the parts deriving their nerves from the destroyed portions, acting in a manner analogous to ligatures, narrowing the boundaries ries of the circulation. He concludes that each portion of the spinal marrow communicates as much nervous influence to the heart, as is necessary to preserve the circulation in the parts to which the nerves from that portion are distributed. After eight unsuccessful attempts, he at length succeeded in demonstrating this fact, by keeping the thorax and anterior extremities of a rabbit alive, after having removed the other parts of the body. He began by passing a ligature round the aorta, immediately below the cœliac artery, and another round the vena cava near the liver; these ligatures were not at first tightened. The carotid arteries and both jugular veins were then tied, and an opening made into the trachea for the purpose of keeping up artificial respiration. He then divided the spinal marrow near the occiput, and began to inflate the lungs. After continuing the inflation for three or four minutes, he detached the trachea from the larynx, and removed the head. After continuing the inflation for three or four minutes longer, he tied the aorta and vena cava: then resumed the inflation, next removed the whole posterior part of the body below the ligatures. He then took away the stomach and liver, taking care to restrain the hæmorrhage. He then kept up inflation as long as the signs of life continued. He repeated this experiment several times, destroying, in some instances, the remainder of the cervical portion, and a part of the dorsal portion of the marrow; yet life was preserved by the two posterior thirds of the dorsal portion. As the author has defined life to consist in sensation and motion, he considers the animal or part of an animal which has lost these properties as dead. He concludes this memoir with some observations on the impossibility of restoring an animal to life which has experienced universal death. This impossibility arises from the total annihilation of the influence which disposes the heart to contract, so that no means remain to carry on the circulation through the nervous organs. But what he calls a partial resurrection may be easily effected. If we tie the descending aorta, the inferior portion of the spinal marrow is deprived of blood, and every sign of life ceases in the lower extremity. But the remaining portions being sufficient to keep up the heart's action, when the aorta is untied, the circulation again commences in the portion before obstructed, and the signs of life return.

Mons. le Gallois draws a great number of conclusions from the experiments detailed in these memoirs, but the most important inferences are summed up by the Committee of the French Institute in their report. They are as follows:

1. That the principle of the motions of inspiration has its seat near that portion of the spinal marrow which gives origin to the eighth pair of nerves. 2. That

2. That the principle which animates each part of the body, resides in that part of the spinal marrow, from which its nerves originate.

3. That it is from the spinal marrow that the heart derives the principle of its life and of its motions: but from the whole marrow, and not from any particular part of it.

4. That the great sympathetic nerve arises from the spinal marrow, and that the particular character of this nerve is to place the parts to which it is distributed under the immediate influence of the whole nervous power.

The third memoir consists of an enquiry into the manner in which death takes place after the division of the par vagum. We hope to be able to give some account of it in our next Number.

---

*Observations sur le Nature & le Traitment des Maladies du Foie; par* Antoine Portal. 1813. pp. 608.

The known talents and industry of this venerable practitioner, especially in the department of pathological anatomy, naturally excited the most sanguine expecations in our minds; nor have we been disappointed in the perusal of the present volume. A judicious treatise on these complaints has long been a desideratum with us; and we may venture to affirm this will be read with considerable advantage by the admirer of pathological researches, and will tend in a great measure to supply the deficiency we have so long had reason to lament.

From an extensive work, possessing nearly uniform claims upon the attention of the medical world, it will hardly be possible to give a complete idea of its execution by the usual analysis. The limited space allotted to articles of this description, will only allow us to enumerate the general contents, and to give short selections to illustrate its execution.

The first part contains an account of the diseases of the liver, whose seat in this organ is generally admitted. Eight chapters are devoted to this inquiry, under the following arrangement.

Pains of the liver—increase or diminution of size, obstructions, induration or preternatural softness, of this organ—jaundice—hepatic colic—bilious fever—bilious colic—inflammation of the liver, and its consequences—and, lastly, hepatic phthisis.

Under the second and remaining part is included the state of the liver in those diseases which are generally supposed to be situated in other organs, the greater part terminating in *hepatic phthisis.*

The following is the arrangement of this division of the work:

The state of the liver in some catarrhal affections—in eruptive diseases—scrofulous complaints—venereal disease—scurvy—rheumatism and gout—rickets—fevers: intermittent, continued, remittent, &c. and in the hepatic phthisis which, more or less generally, attends these complaints—in certain dropsies—after mental affections and violent pains—difficulty of breathing—palpitations of the heart, syncopes, and angina pectoris—in nausea and dyspepsia, with various evacuations from the stomach and bowels—bilious vomitings—vomitings of blood—melœna—some diarrhœas—dysentery—purulent evacuations from the stomach and bowels—hepatirrhœa—cholera morbus and iliac passion—and, lastly, the state of this viscus after blows on different parts of the body.

The author, in pursuing the object he has in view, has judiciously brought into one focus a collection of cases, illustrating the general principles he inculcates; and these consist of selections from the most valuable pathological writers, which are followed by such as actually occurred in his own observation, first giving the fatal instances, with their dissections, and afterwards those which yielded to the remedies employed. After the particular examples of the several morbid alterations of this organ, the whole is summed up, and the inferences deduced from them are placed under the general head of Remarks. In giving specimens for the purpose of illustrating the plan of the work, we shall take the first which present themselves, under the head Pains of the Liver.

Case 1.—A man, 36 years of age, who complained a long time of pain in the right hypochondrium, fell into a low fever. He wasted away, and the skin became of a yellow colour. He lost his appetite, and experienced intense thirst, A tumour in the right hypochondriac softened: it was evidently a collection of matter ready to burst: it was opened, and a considerable quantity of pus flowed through the wound, the smell of which was so intolerable that the assistants could scarcely remain in the room. He died on the seventh day from the operation. On dissection, the belly was filled with pus, the omentum was destroyed, and the peritoneum had begun to putrify. The intestines were blackish, or of a leaden colour. The liver was large and hard, and a gangrenous ulceration was found on the right side.—*Forestus.*

E. S. experienced acute pains in the right hypochondriac region for several years, which were supposed to arise from dyspepsia, and were sometimes imagined to be seated in the liver, liver, and at others in the stomach. She took a variety of remedies without benefit. Her complexion was sallow; her urine red; and the stools were clay-coloured. The lower extremities were œdematous, and especially the right. The side in the neighbourhood of the right kidney was extremely painful. The urine was always muddy, and sometimes red as blood. The swelling of the extremities increased, with slight œdema of the belly; the respiration became short, but the pulse continued natural; and finally she died. On opening the body, the liver was found to be the real seat of the complaint, as the author had before affirmed, contrary to the opinion of some physicians in attendance with him, who thought the right kidney diseased. The liver was of its usual size, but hard, and greyish in several parts. The gall bladder was absolutely obliterated, as was the cystic duct, the coats of which were converted into a cartilaginous substance. The ductus coledochus was contracted; the lungs hard and swollen; but the remaining viscera sound.

Case 1st successfully treated.—A student in medicine, 23 years of age, of a studious disposition, wasted considerably, although he preserved his appetite. A *dry cough* arose, which distressed him, particularly *after dinner, with a pain in the right side of the chest, incommoding respiration.* His chest appearing to be the seat of the disease, and thinking it a case of incipient phthisis, the author advised emollient broths, with milk diet; but a jaundice soon appeared, and he was again consulted. The belly was examined, and the liver found tumid, especially in the epigastric region. He complained of frequent colics. He had an itching of the skin, with eruptions of an anomalous kind, which disappeared without particular treatment; and his urine was of a higher colour than usual. There appeared then no doubt of the liver being the seat of the disease, though the lungs could not positively be pronounced to be in a healthy state. But as there was no expectoration or spitting of blood, the treatment proceeded solely on the former supposition.

An emetic was administered. This was followed by saponaceous pills, with bitter extracts, and infusion of soapwort and hops. The symptoms gradually diminished, and the patient recovered. The author observes, this case, among many others, proves that *pain in the chest* may arise from diseased liver.

In the *Remarks* belonging to this division, the author commences by an attempt to shew that the liver itself is capable of sensation, contrary to an opinion formerly entertained that the pain was seated in the membranes.

Hepatic pains are principally felt in the epigastric region,

 beneath beneath the zyphoid cartilage, whence they extend into the right hypochondrium, under the false ribs, to the kidney of the same side. Sometimes they are felt in the left hypochondrium, but are more frequently referred to the stomach, the greater part of the inferior and internal surface of the liver being contiguous to it. It is the more important to make these observations, because it is generally supposed to be seated only in the right side, which in reality is seldom the case; whereas they frequently exist in the horizontal or left portion over the stomach, which Ferrein has very properly remarked. However I speak only of the generality of cases. There are individuals who have suffered pain a considerable time in the *region of the gall bladder*, from calculi contained in it; and in inflammations and other diseases of the liver, pain may unquestionably be felt throughout its whole substance. Simple obstructions are often accompanied by pains which in some is felt in particular parts, in others is more generally diffused; sometimes they are concentrated in this viscus, and often they are extended to neighbouring organs.

Of all the causes of pains, the most frequent is the bile itself, or biliary calculi, which gives rise to the term hepatic colic, a disease described in all ages, and which possesses a characteristic mark, which we shall mention hereafter.

To the pains in the stomach consequent to *diseased liver*, should be added those affections of the heart, præcordia, and diaphragm—cardialgiæ, gashalgiæ, gastrodyniæ, &c. How difficult do we not find it to distinguish the real seat of these pains, as well as of affections of the spleen, pancreas, kidney, &c. We must, therefore, in order to establish a correct diagnosis, make a comparative analysis of the symptoms which belong to the injuries of each organ respectively. If, for example, there is vomiting, there can be no doubt but the stomach is primarily or secondarily affected. If jaundice is present, the liver will be fixed upon as the seat of the complaint, although it is very possible for the latter to exist without the former. If there is suppression of urine, we naturally look to the kidneys; in retention, the bladder. If the patient has the *risu sardonicus*, with pains in the shoulders and arms, the liver and the diaphragm are probably the parts affected. From the aggregate of the symptoms alone we must draw our conclusion; but happily, in cases of danger, our practice is guided not so much by the nature and intensity of the disease, as by the presence or absence of fever.

When the pain is very violent, the seat of the disease cannot be correctly ascertained by pressure on the external parts; it is only when it is moderate that we can distinguish by by this means whether it be in the stomach or liver. If in the latter, it is generally felt immediately below the zephoid cartilage, and pressure gives to the patient a sensation as if the part was bruised or sore; if in the stomach, it is generally more acute, and lower down. These remarks are due to Ferrein, but, we repeat it, they are only true when the pain is not very violent; for then they are not limited, sometimes passing into the left hypochondrium, but oftener into the right, where they can generally be excited by pressing with the fingers. Ferrein assures us also that when the pains arise from the stomach, and from a collection of bad humours in it, the pulse is intermittent. This skilful anatomist says, that he made these remarks before Nihill, an English physician, who wrote on the pulse many years ago. Baillon, who thought that melancholia had its principal seat in the liver, observed that patients in this disease, instead of experiencing pain in the hypochondrium, referred it to the chest.*

Sometimes pain from this source is felt in the heart, and still oftener in the stomach; sometimes in the left hypochondrium, in the spleen; at others in the umbilicus, small intestines, kidneys, and especially in the right one. These variations arise from the operation of nervous sympathy. Many facts prove the fallacy of the opinion, *ubi dolor ibi morbi sedes.* We have seen, in one of the cases quoted from Valsalva, that a fatal disease of the lungs was referred to the liver, and how easy would it be to multiply examples of this kind?† The spleen has been thought diseased when the liver

* Baillon Consil. Med. T. Q. p. 131, Annot.

† The case referred to is the following:—A patient had, three years previous to the present illness, an acute fever, which terminated critically by suppuration in the glands. After this he had an intermittent fever, which teased him a long time, and at last he recovered. He remained pale and thin. At times he experienced great difficulty of breathing. His sleep was disturbed; his urine red, with high fever. He complained of pain beneath the *right false ribs, and below the zyphoid cartilage, which was increased on pressure.* At the commencement of these symptoms he had *vomiting and purging, a cough,* at first *moist,* but in a few days *dry* and obstinate, with inability to lie on either side. He experienced also an acute sensation of heat in the back and right kidney. The pulse was quick, frequent, weak, intermitting, and irregular. Valsalva was at first doubtful respecting the seat of the disorder, but as no pain had been felt in the chest, and the patient always held his hand in the neighbourhood of the liver, he considered this viscus to be the part affected. The patient grew rapidly worse: the difficulty of breathing was extreme; the pulse became daily more weakened; and he died on the seventh the liver was affected; the same mistake has been made with the right kidney, and vice versa. Acute pain in the umbilicus, produced by calculi in the gall bladder and biliary ducts, has been referred to worms in the intestines; and Morgagni relates a case of *ulcerated cancer* of the mesentery, mistaken for disease of the liver.

It is ascertained that itching at the end of the nose, with or without the *risus sardonicus*, which have been generally ascribed to worms, have been occasioned by an affection of the liver and diaphragm. The pain at the lower part of the neck above the right shoulder, extending down the arm, has been also proved to be the effect of this disease. In some cases where the left lobe was principally affected, the patients have experienced pain in the left shoulder, no doubt from the affection of the left diaphragmatic nerve. In other cases, pain has been felt in both arms and shoulders, probably from a greater extension of the disease. As the affection of the shoulders and arms are also felt in angina pectoris, we can readily see how the diseases of the diaphragmatic nerves will produce the same in diseases of the liver.

Spasmodic contractions of the diaphragm are often occasioned by diseases of the liver. This was previously remarked by Fernelius. The sensation is described as though the patient is bound with a cord—*Quasi fune stringeretur.*

Hepatic pains, though obscure, if of long continuance, naturally lead to the suspicion of morbid alteration in the structure of the liver. It is necessary to prevent, if possible, this change from taking place, since, if once confirmed, the disease is for the most part incurable. Inflammation is a frequent consequence, and the termination of this is well known. With a view, therefore, both to our prognosis and treatment, we must be particularly attentive to the nature of this pain; we must inquire whether it be acute or otherwise, whether temporary, durable, or periodical; fixed or wandering; and whether it be accompanied with alarming symptoms or not.

---

day. On dissection, all the viscera of the abdomen were sound, excepting the kidney, whose size was four times larger than usual. In the left cavity of the chest were found two pints of limpid fluid; in the right the fluid was somewhat thicker, with shreds of coagulable lymph floating in it. The lungs did not adhere to the pleura, but the right, without being increased in size, was indurated by previous inflammation. The pericardium contained more fluid in its cavity than usual. The right auricle and ventricle of the heart, independently of polypous concretion, which they contained, were full of coagulated blood. In the left ventricle there was also coagulated blood, but in smaller quantity.

A consideration

A consideration of the cause will materially assist us in these respects. Pain may be very acute in melancholic or hysterical subjects, and in irritable children, without very serious consequences. It is sometimes extremely acute, with vomitings and convulsive motions, in those who have biliary calculi. Nevertheless, if we touch or lightly compress the part in pain, we do not occasion the same increase of the distress as if it arose from any other cause; and especially if from inflammation.

The most acute pains from biliary calculi cease, as it were by a charm, when the concretions are passed into the duodenum. They may then in general be considered to be free from danger, however intense. On the contrary, the pain is so obscure in scrofulous subjects, that matter may be formed to a considerable extent with scarcely a suspicion of the nature of the disease, as we have frequently discovered on dissection after death.

When combined with fever, it always requires the greatest attention, as it indicates an inflammatory disposition; and as, in scrofulous affections, this rarely takes place until the last stages, we shall be able, from the knowledge of this circumstance, to form a correct estimate of the degree of danger with which the symptom is attended, from a consideration of the particular constitution of the patient.

(To be continued.)

